# Purification of angiotensin converting enzyme inhibitory peptides and antihypertensive effect generated from Indonesian traditional fermented beef (Cangkuk)

**DOI:** 10.5713/ab.23.0433

**Published:** 2024-05-07

**Authors:** Irdha Mirdhayati, Wieda Nurwidada Haritsah Zain, Abdul Fatah, Issei Yokoyama, Keizo Arihara

**Affiliations:** 1 Department of Animal Science, Faculty of Agriculture and Animal Science, Universitas Islam Negeri Sultan Syarif Kasim Riau, Pekanbaru, 28293, Indonesia; 2 Department of Animal Science, School of Veterinary Medicine, Kitasato University, Towada-shi, 034-8628, Japan

**Keywords:** ACE-inhibitory Activity, Antihypertensive Effect, Cangkuk, Peptide Purification, Spontaneously Hypertensive Rats, Traditional Fermented Beef

## Abstract

**Objective:**

Traditional fermented meat products can be considered a source of bioactive peptides. Cangkuk, a traditional Indonesian fermented beef product is one source of angiotensin converting enzyme (ACE) inhibitory peptides. This study aimed to identify ACE-inhibitory peptides from Cangkuk and analyze their antihypertensive effects.

**Methods:**

The water-soluble fraction of Cangkuk was fractionated to obtain ACE-inhibitory peptides using an ethanol solvent at several concentrations and solid-phase extraction with an OASIS HLB cartridge followed by purification with reversed-phase high-performance liquid chromatography (RP-HPLC). HPLC-MS was used to identify target peptides, followed by automatic protein sequencer analysis to detect peptide sequences. Antihypertensive effects were analyzed on the water-soluble fraction and synthesized peptides. The animal model comprised 14-16-week-old male spontaneously hypertensive rats (SHRs) (~320 g average body weight) with mean systolic blood pressures (SBPs) higher than 190 mmHg. All oral doses of peptides were 1 mL in volume. Distilled water was used as a control. The antihypertensive activities of the sample and control were observed by measuring the SBP at 0, 2, 4, 6, 8 and 24 h after oral administration.

**Results:**

Two sequences of ACE inhibitory peptides were found, EAPLNPKANR (IC_50_ value of 44.6 μmol/L) and IVG (IC_50_ value of 97.3 μmol/L). The water-soluble fraction demonstrated an antihypertensive effect on SHRs after oral administration at 100 mg/kg body weight, maximally lowering the SBP by 14.9 mmHg 8 h after administration. The tripeptide IVG showed the highest reduction of SBP, 24.76±2.1 mmHg 8 h after administration. The decapeptide EAPLNPKANR showed the highest reduction of SBP, 21.0±1.9 mmHg, 8 h after administration. All the samples differed significantly from the control (p<0.01).

**Conclusion:**

Cangkuk has potential as a functional food ingredient acting as an antihypertensive agent.

## INTRODUCTION

Hypertension is a considerable risk factor for cardiovascular morbidity and mortality [1]. As one of the main causes of premature death worldwide, approximately 1.13 billion people globally have hypertension [2]. Treatment to reduce high blood pressure generally uses synthetic drugs, but the safety of these compounds remains a concern [3]. Antihypertensive agents from natural sources, particularly angiotensin-converting enzyme (ACE) inhibitory peptides from animal protein, have attracted the attention of many researchers owing to its fewer adverse side effects and greater safety [4,5].

Meat and meat products are excellent sources of ACE-inhibitory peptides [6]. ACE-inhibitory peptides from animal proteins can be produced by several methods, including enzymatic hydrolysis and fermentation [7]. Several potent ACE-inhibitory peptides from meat can be generated from enzymatic hydrolysis using commercial protease, *e.g*., the alkaline-AK-pepsin hydrolyzed peptide Leu-Ile-Val-Gly-Ile-Ile-Arg-Cys-Val from beef myofibrillar protein, which had an antihypertensive effect in SHRs [8]. The pepsin hydrolyzed peptide Lys-Arg-Val-Ile-Gln-Try and the Val-Lys-Ala-Gly-Ph sequence from porcine skeletal myosin B showed antihypertensive effects *in vivo* [9]. ACE-inhibitory peptides derived from traditional fermented meat with antihypertensive effects have not been widely reported. This is an important area to explore for discovery of alternative natural antihypertensive agents.

Many sources of bioactive peptides from food fermentations have been explored. Particularly, ACE inhibitory peptides can be generated from fermented milk and milk products [10]. Popular fermented meat products, such as many types of sausage and dry-cured ham, have been found to contain ACE inhibitory peptides that have antihypertensive effects [11,12].

According to a previous study, “Cangkuk” is made from fresh beef to which bamboo shoots, rice and salt are added. The treatment evaluated in the current research was addition of bamboo shoots with different preparation methods (chopped, ground, and extracted) and varied meat:bamboo shoot ratios (1:0.75, 1:1, and 1:1.25). Then, these preparations were fermented under anaerobic conditions at room temperature for seven days. It was reported that all treatments had ACE-inhibitory activities ranging from 43% to 80%. The best ACE-inhibitory activity (80%) was shown by Cangkuk, which was processed by adding ground bamboo shoots and meat at a ratio of 1:0.75 [13]. Traditional fermented foods from Asia have been reported to provide enhanced nutritional content, digestibility, and microbial stability. Other traditional fermented meats have been studied, *e.g*., “Nham” from Thailand, made of pork meat, garlic, salt and rice, and “Nem-cua” from Vietnam, made of pork meat, salt and cooked rice [14]. Unfortunately, no study has reported the bioactivity of traditional fermented meats, especially their antihypertensive activities.

Traditional fermented meat has a high potential as a new source of ACE-inhibitory peptides due to the use of endogenous enzymes associated with lactic acid bacteria in fermentations that hydrolyze meat proteins [15]. The advantages of microbial fermentation are that it can generate bioactive peptides more economically and cost-effectively than enzymatic hydrolysis, and the possibility of using natural ingredients as a growth medium for lactic acid bacteria [16]. This study aimed to purify and identify ACE inhibitory peptides from traditional Indonesian fermented meat and investigate their antihypertensive effects in spontaneously hypertensive rats (SHRs).

## MATERIALS AND METHODS

### Materials

The raw materials used in processing Cangkuk were fresh beef meat (brisket) from male Bali cattle, aged 2.5 to 3 years, purchased from the Animal Slaughtering House in Pekanbaru City. Green bamboo shoots were harvested from local farms. The additional ingredients were cooked rice, salt and distilled water. Angiotensin converting enzyme (from rabbit lung) and the substrate, hippuryl-L-histidyl-leucine, were purchased from Sigma-Aldrich Co. (St. Louis, MO, USA). All the chemicals and reagents used were of analytical grade.

### Methods

**Figure 1:**
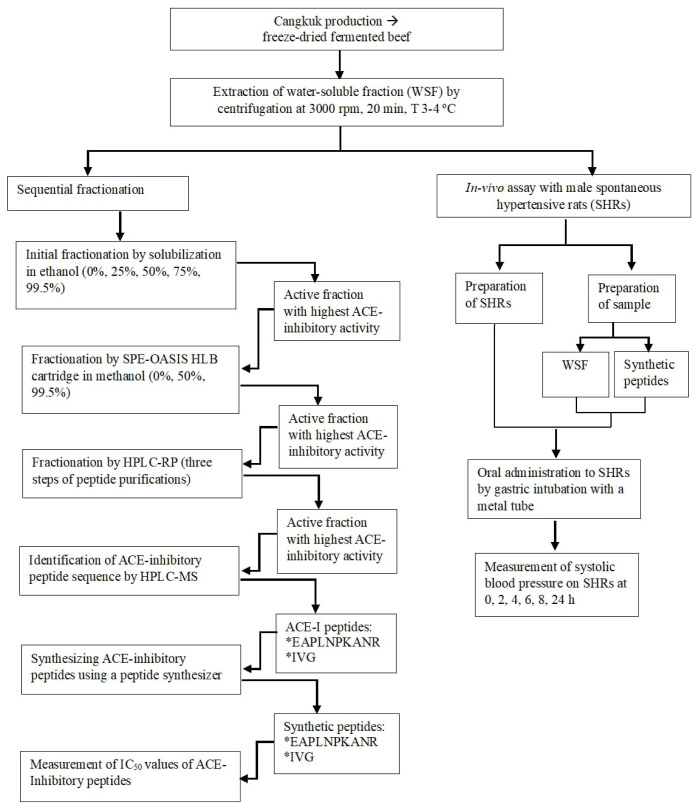
Flowchart depicting experimental purification ACE-inhibitory peptides from fermented beef, Cangkuk. ACE, angiotensin converting enzyme.

This experimental research consisted of two stages. The first was purification of ACE-inhibitory peptides from Cangkuk. This stage included production and extraction of Cangkuk, sequential fractionation of the most active fraction, analysis of ACE-inhibitory activity on each fractionation step, identification of ACE-inhibitory peptides, and chemical synthesis of ACE-inhibitory peptides. The second stage involved analysis of the antihypertensive effects of the water-soluble fractions and ACE inhibitory peptides from Cangkuk. This stage involved preparation of animal models, preparation of samples, oral administration to SHRs, and measurement of systolic blood pressure (SBP) of the rats. A flowchart depicting this experimental methodology is shown in Figure 1.

### Production of Cangkuk

Cangkuk was processed as described by Salahuddin [17] and modified with bamboo shoots in its preparation. Fresh beef meat was separated from its fat and connective tissue and then washed with distilled water. The meat was cut into small pieces, approximately 5×5×2 cm^3^. Cangkuk was initially prepared with 300 g of small cuts of beef meat and ground bamboo shoots (225 g of bamboo shoots and 225 mL of distilled water, homogenized for 3 min in a blender) and put into polypropylene plastic boxes. Salt and cooked rice were added at 1% w/w of the total beef meat and bamboo shoot weight. All the ingredients were mixed to homogeneity. Then, the plastic box was tightly covered and sealed with tape to ensure anaerobic conditions. The fermentation was allowed to proceed for seven days under a dark condition. The temperature and humidity were 25°C±2°C and 70%±5%, respectively. After the fermentation, fermented meat was separated from the other ingredients and ground. The ground fermented meat was freeze-dried (Buchi Lyovapor L-200; Buchi, Flawil, Switzerland). Dried Cangkuk was then packed in laminated aluminum-plastic bags and stored in a freezer at −20°C prior to the extraction step.

### Cangkuk extraction

Extraction of a water-soluble fraction of Cangkuk was done as reported by Phadke et al [18] who examined preparation of aqueous extract from fermented fish. An amount of 40 g of dried Cangkuk and 360 mL of distilled water were mixed using a homogenizer (magic bullet) (Nissei AM_3/Ace Homogenizer; Nihonseiki Kaisha Ltd., Osaka, Japan) for 30 seconds. This process was repeated three times. The homogenous solution was centrifuged at 3,000 rpm for 20 min at a temperature of 3°C to 4°C. The supernatant was filtered through a No. 2 Advantec filter paper (Toyo Roshi Kaisha, Ltd., Tokyo, Japan) to obtain a water-soluble fraction. The retentate was extracted again by adding 200 mL of distilled water and continuing the extraction process with similar steps to obtain a water soluble fraction. The water-soluble fraction of Cangkuk was freeze-dried (Eyela, Tokyo Rikakikai C., Tokyo, Japan) and stored in a freezer at −20°C prior to purification and animal assay.

### Purification and identification of ACE-inhibitory peptides

Purification of ACE-inhibitory peptides in this study was done using several levels of fractionation. The first employed an ethanol solvent, while the second used a solid phase extraction-HLB cartridge with a methanol solvent. The third level used a high performance liquid chromatography-reverse phase column with a type C-18 column. The complete details of each fractionation step are as follows.

#### Fractionation using ethyl alcohol

A mass of 2.01 g of the water-soluble fraction of Cangkuk was dissolved in 50 mL of 99.5% ethanol and stirred for 30 min. The solution was then filtered using No. 2 Advantec filter paper and the collected filtrate was subjected to evaporation in a rotary evaporator (Buchi Rotavor R-124; Buchi, Switzerland) at 50°C. Retentate on the filter paper was rinsed again with 50 mL of 75% ethanol and the filtrate collected. This step was conducted in sequence on the samples by adding 50 mL of 50% ethanol, 25% ethanol, and 0% ethanol, respectively. Each ethanol fraction filtrate was freeze-dried and analyzed for ACE-inhibitory activity. The most active portion was fractionated using a hydrophilic lipophilic balance (HLB) cartridge.

#### Fractionation by OASIS HLB extraction cartridge

The most active ethanol fraction (50%) was dissolved in 100 mL of distilled water and poured into an OASIS HLB cartridge (Waters, Dublin, Ireland). An amount of 100 mL of the eluate was collected and concentrated using a rotary evaporator with a 0% methanol fraction. In subsequent steps, 100 mL of a 50% methanol fraction was poured into a cartridge. Then, the eluate was collected and concentrated. A similar procedure was followed for a 100% methanol fraction. Each eluate from three methanol fractions was freeze-dried and analyzed for ACE inhibitory activity. The most active portion was fractionated using reverse phase-high performance liquid chromatography (RP-HPLC).

#### Fractionation by HPLC with a reverse-phase column

The fractionation steps using RP-HPLC were conducted as described by Mirdhayati et al [19] with slight modifications in the flow rate. The most active fraction from HLB extraction was dissolved in 1.5 mL of distilled water and further fractionated with high-performance liquid chromatography in a reverse-phase mode, HPLC-RP (column CAPCELL PAK C18, MG II 4.6 MM ID X 150 MM; Shiseido Co., Tokyo, Japan). Elution was performed using a linear gradient system from solvent A (distilled water [Millipore] containing 0.05% formic acid) to solvent B (acetonitrile containing 0.05% formic acid) at a flow rate of 0.8 mL/min at 40°C. Absorbance was measured (HPLC-RP; Prominence LC20AT; Shimadzu, Kyoto, Japan) at 215 nm. Each fraction was analyzed for ACE inhibitory activity (first HPLC run). The active portion was subsequently fractionated using HPLC-RP employing a similar system and analyzed for ACE-inhibitory activity (second HPLC run). The active fraction from the second HPLC run was dissolved in distilled water and re-fractionated using HPLC-RP (loaded on column X Bridge BEH 130, C18, size 2.1 mm ID×10 mm [3.5 μm]); Waters Co., Ireland). Elution was performed with a linear gradient (solvent A, 10 mM ammonium hydrogen carbonate in distilled water [Millipore]; solvent B, acetonitrile) at a flow rate of 0.4 mL/min at 40°C (third HPLC run). Each fraction was analyzed for ACE-inhibitory activity. The molecular weight from two active fractions was confirmed using an HPLC-MS 2020 System (Shimadzu-Biotech, Kyoto, Japan). Two active fractions were collected and concentrated, and their amino acid sequences identified.

### Identification of purified peptides

The two active fractions, identified by their molecular weights with an HPLC-MS 2020 system, were used to determine their amino acid sequences employing an Edman degradation method with an automatic protein sequencer (PPSQ-31A; Shimadzu-Biotech, Japan), equipped with an online system for PTH amino acids.

### Chemical synthesis of identified peptides

Two sequences of ACE-inhibitory peptides (IVG and EAPLNPKANR) were synthesized using a solid-phase method with a peptide synthesizer (BEX Company, Tokyo, Japan). Peptides with a purity of 80% were determined using HPLC. The IC_50_ values of these synthetic peptides were measured for ACE-inhibitory activity and administered to SHRs to examine their antihypertensive effects.

### Analysis of ACE-inhibitory activity

ACE-inhibitory activity was analyzed according to the method of Cushman and Cheung [20] with modifications by Arihara et al [21]. This assay is based on liberation of hippuric acid from Hip-His-Leu catalyzed by ACE. A sample solution (15 μL) was mixed with 125 μL of a 100 mM sodium borate buffer (pH 8.3) containing 7.6 mM Hyp-Hys-Leu and 608 mM NaCl followed by pre-incubation for 5 min at 37°C. The reaction was initiated by adding 50 μL of ACE dissolved in distilled water, and the mixture was incubated for 30 min at 37°C. The reaction was stopped by adding 125 μL of 1 N HCl. The hippuric acid liberated by ACE was extracted by adding 750 μL of ethyl acetate to the mixture. Hippuric acid was dissolved with 1 mL of distilled water and its concentration spectrophotometrically determined at 228 nm. ACE inhibitory activity was calculated as:


Inhibitory activity (%)=[(C-A)/(C-B)]×100

where A, absorbance of the sample reaction; B, absorbance of the blank; and C, absorbance of the control (distilled water).

### Antihypertensive activity

#### Animal preparation

All procedures involving animals were performed in accordance with “the animal handling protocol approval” which is issued by the local animal care committee of the School of Veterinary Medicine and Animal Science of Kitasato University, Japan (Approval No. 20-085). Six 4-week-old male SHRs were purchased from Charles River Japan, Inc. (Yokohama, Japan), and were housed in cages on a cycle of 12 h light and 12 h dark periods (8:00 to 20:00). There were six cages, and each cage consisted of three rats. The temperature and humidity in the cages were controlled at 23°C±2°C and 50%±10%, respectively. Rats were fed a standard diet (CE-2; Clea Japan, Inc., Tokyo, Japan), and water was available *ad libitum*. Eventually, to evaluate the antihypertensive effect of peptides, 14-week-old SHRs (body weight [BW] mean ~320 g, n = 6, SBP mean greater than 190 mmHg) were used.

#### Sample preparation for SHR experiments

There were two simple trials to test the antihypertensive effects of Cangkuk. The first experiment tested was the antihypertensive effect of a water-soluble Cangkuk fraction. As the control treatment was administration of distilled water, the sample treatments were dosed with water-soluble Cangkuk fractions at 100 mg/kg and 1,000 mg/kg of rat BW. A second trial was done to test the antihypertensive effects of ACE-inhibitory peptides from Cangkuk. As the control treatment was distilled water, the sample treatments were dosed at 0.35 mg of IVG and EAPLNPKANR peptides, respectively. All the volumes for oral administration were 1 mL.

#### Measurement of blood pressure

Rats divided in three group according to sample treatment. The blood pressure measurements of SHRs were done according to Mirdhayati et al [19]. Rats were each administered a solution sample by gastric intubation with a metal tube (Natsume Seisakusho Co., Tokyo, Japan). The SBP of each SHR was measured using the tail-cuff method with a programmed electrosphygmomanometer (BP-98; Softron Co., Tokyo, Japan). The antihypertensive activities of the sample and control were observed by measuring the SBP at 0, 2, 4, 6, 8 and 24 h after oral administration. Data were expressed as mean SBP values on six SHRs at each time. Significant differences from the control were evaluated. The measurements were made in triplicate, and average values were reported.

### Statistical analysis

Statistical analysis was applied on the antihypertensive test data using SHRs. Changes in SBP were determined by the difference between SBPs before and after administration. Data are expressed as mean values and standard errors of the mean. The differences in SBP between control and sample groups were statistically analyzed using two-way repeated-measures analysis of variance with Dunnett’s test.

## RESULTS

### Purification and identification of ACE-inhibitory peptides

The ACE-inhibitory peptides of fermented beef (Cangkuk) were initially fractionated using ethanol solutions at concentrations of 0%, 25%, 50%, 75%, and 100%. ACE inhibitory activity was highest (±83.28%) in the 50% ethanol fraction, known as the active fraction. Then, the active fraction was again fractionated using a solid-phase extraction (OASIS-HLB Cartridge) at three methanol concentrations (0%, 50%, and 100%). The 50% methanol fraction had the highest ACE inhibitory activity (±51.28%).

**Figure 2:**
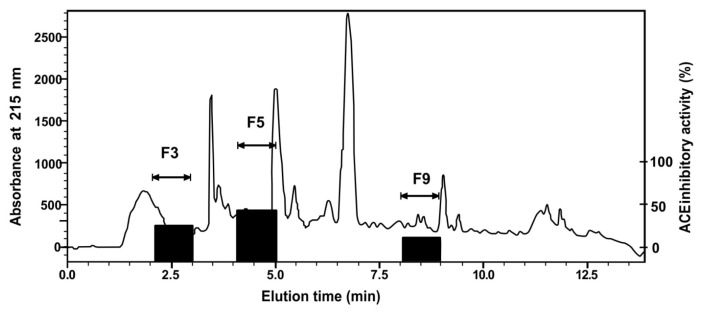
Fractionation of fermented beef (Cangkuk) using reversed-phase high-performance liquid chromatography (RP-HPLC) (first HPLC run). Elution time 0–10 min, 1 min interval fractionation. Fractions were collected and assayed for ACE-inhibitory activity. Fraction 5 had an elution time of 4.0 to 5.0 min. ACE, angiotensin converting enzyme.

**Figure 3:**
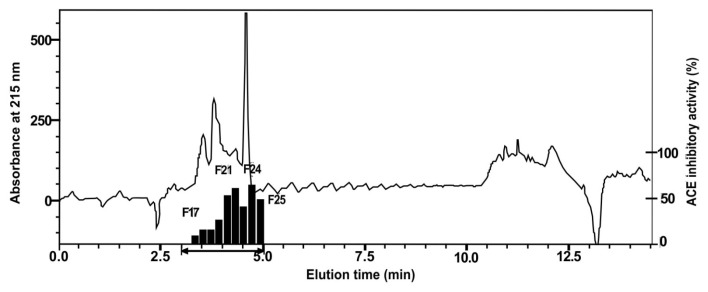
Fractionation of fermented beef (Cangkuk) using reversed-phase high-performance liquid chromatography (RP-HPLC) (second HPLC run). The active fraction had an elution time of 3–5 min, with 0.2 min interval fractionation. Fractions were collected and assayed for ACE-inhibitory activity. The active fractions were F22 and F24. ACE, angiotensin converting enzyme.

The most active fraction from the SPE OASIS-HLB cartridge was further purified using reversed-phase high-performance liquid chromatography (RP-HPLC). The resulting chromatogram (Figure 2) shows three active fractions. They are Fraction 3 (elution time 2.0 to 3.0 min), Fraction 5 (elution time 4.0 to 5.0 min), and Fraction 9 (elution time 8.0 to 9.0 min) with ACE-inhibitory activities of 27.7%, 42.2%, and 18.6%, respectively. Fraction 5, with the highest ACE-inhibitory activity, was further purified using a second HPLC run. The active fractions were F22 and F24, with ACE-inhibitory activities of 62.7% and 66.9%, respectively (chromatogram in Figure 3).

The active fraction, F22, was further purified using a different column and procedure (third HPLC run), producing two active fractions, Fraction 8 and Fraction 10, with ACE-inhibitory activities of 35.9% and 39.3%, respectively. The molecular weights of these fractions were identified using an HPLC MS 2020 system. Fractions 8 and 10 had molecular weights of 1,109.70 Da and 288.10 Da, respectively. Then, Fractions 8 and 10 were collected, concentrated, and subjected to amino acid sequencing using an automatic protein sequencer. The amino acid sequence of these two active fractions is shown in Table 1.

### Antihypertensive activity

**Figure 4:**
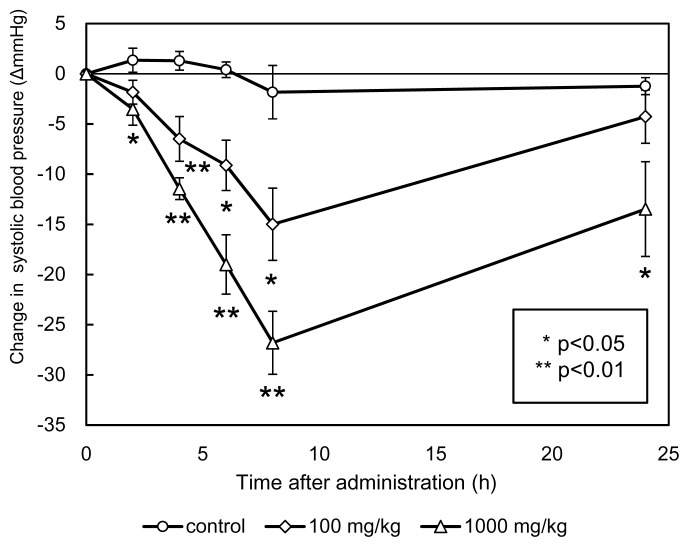
Antihypertensive activities of single oral administration of fermented beef (Cangkuk) at 100 mg/kg body weight and 1,000 mg/kg body weight. Each point indicates the mean of systolic blood pressure of six SHRs, and the vertical bars represent the standard error. Distilled water was used as a control. Significant differences from the control at each time: * p<0.05, ** p<0.01.

The changes in SBP of SHRs after a single oral administration of fermented beef at concentrations of 100 mg/kg BW and 1,000 mg/kg BW are shown in Figure 4. Distilled water was used as a control, where the SBP of SHRs did not change significantly during 24 h of observation. As shown in Figure 4, the SBP of SHRs after administering 100 mg/kg BW of fermented beef was lower than that of the control group between the 2 h to 24 h observations (p<0.05). The reduction in SBP was 14.9±3.6 mmHg 8 h after administration. A similar pattern was observed in the SBPs of SHRs after administering 1,000 mg/kg BW of fermented beef (Cangkuk). The reduction was more significant due to higher levels of fermented beef peptides given. The highest reduction of SBP was 26.8±3.1 mmHg, which occurred 8 h after administration compared to the control (p<0.05). SBPs of SHRs administered at 1,000 mg/kg BW of fermented beef were significantly lower than that of the control group after 24 h (p<0.05), but not significantly different for SHRs administered with 100 mg/kg BW of fermented beef.

**Figure 5:**
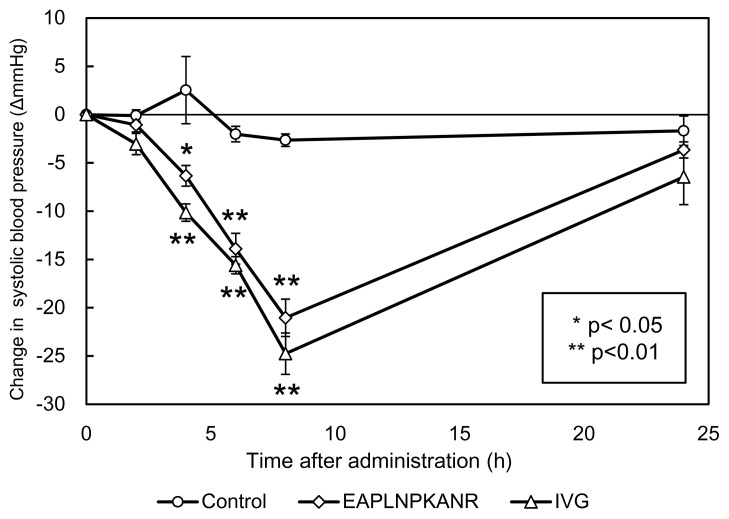
Antihypertensive activities of single oral administration of synthesized peptides (EAPLNPKANR and IVG) at a concentration of 0.35 mg/mL distilled water. Each point indicates the mean of systolic blood pressure of six SHRs, and the vertical bars represent the standard error. Distilled water was used as a control. Significant differences from the control at each time: * p<0.05, ** p<0.01.

The change of SBPs of SHRs after a single oral administration of each of the synthesized peptides, EAPLNPKANR and IVG, are shown in Figure 5. Distilled water was used as a control. The SBPs of the SHRs did not change significantly during 24 h of observation. The tripeptide IVG showed the highest reduction in SBP, 24.76±2.1 mmHg 8 h after administration. It was significantly different compared to the control (p<0.01). The decapeptide EAPLNPKANR showed the highest reduction of SBP, 21.0±1.9 mmHg, 8 h after administration and was significantly different than the control (p<0.01). SBPs of SHRs were not different from the control at 2 and 24 h. The tripeptide IVG significantly lowers SHRs blood pressure and to a greater degree than the decapeptide, EAPLNPKANR.

## DISCUSSION

The current study found that a 50% ethanol solution is most effective after initial fractionation of ACE inhibitory peptides from fermented beef. This activity was influenced by the presence of soluble peptides consisting of hydrophilic and hydrophobic amino acids. The 50% ethanol solution is a semi polar solvent that dissolves both polar and non-polar compounds. Hydrophobicity of amino acids in a sequence of bioactive peptides contributes to ACE-inhibitory activity [22]. It has been reported that a 50% ethanol solvent was effective in extracting low molecular weight proteins (peptides) from snakehead fish fillet extract and indicated ACE-inhibitory activity [23].

Further purification of active fractions using an OASIS-HLB cartridge allows purification of peptide compounds with a wide range of polarity. The active fraction obtained from this separation can be used directly for further fractionation *via* RP-HPLC. The 50% methanol fraction is the most active with the highest ACE-inhibitory activity. The amino acid composition of soluble peptides in a 50% methanol fraction affects its ACE-inhibitory activity. Factors determining ACE-inhibitory activity are amino acid composition, propensity to bind hydrophobic amino acids, small peptides, and molecular weights <3,000 Da [24].

In this research, two ACE-inhibitory peptides were identified from the water-soluble extract of Cangkuk with a three-step purification using RP-HPLC, namely EAPLNPKANR (IC_50_ value of 44.6 μM) and IVG (IC_50_ value of 97.3 μM). EAPLNPKANR, is a fragment of the 3rd to 12th amino acid sequence of LTEAPLNPKANREK [25]. LTEAPLNPKANREK is the ACE-inhibitory peptide from goat meat hydrolysate, hydrolyzed by gastrointestinal proteases (pepsin, trypsin, and chymotrypsin) with an IC_50_ value of 120 μM, but its antihypertensive effect has not yet been reported. EAPLNPKANR had higher ACE-inhibitory activity than LTEAPLNPKANREK, the parent peptide. Presumably, the presence of glutamic acid as an N-terminal and a shorter peptide fragment derived from spontaneous fermentation of beef contributes to strong ACE-inhibitory activity.

In this study, IVG was found in traditional fermented beef and had lower ACE-inhibitory activity than IVGRPRHQG (IC_50_ value of 6.2 μM), a nonapeptide sequence of ACE-inhibitory peptides from *Katsuobusi* [26]. The presence of hydrophobic amino acids such as isoleucine, valine, and glycine in the sequence has been associated with ACE-inhibitory activity [27]. Novel ACE inhibitory peptides from venison (game meat) treated with pepsin-trypsin-pancreatin, IKEVTER, demonstrated strong ACE-inhibitory activity due to the presence of isoleucine, lysine, glutamic acid, valine, threonine, and arginine [28]. Furthermore, a novel ACE-inhibitory peptide was obtained, a nonapeptide LIVGIIRCV from beef myofibrillar protein hydrolysate that was hydrolyzed using Alkaline-AK [8]. Fermentation of beef meat by addition of bamboo shoots could generate potent ACE-inhibitory peptides that are comparable to those of controlled enzymatic hydrolysis and thermal processing of meat.

Finding two types of ACE inhibitor peptides in Indonesian fermented meat products (EAPLNPKANR and IVG) is closely related to the activities of microbial enzymes that cleave the peptide bonds of beef protein during fermentation. Lactic acid bacteria are common microorganisms in food fermentations, and their extracellular endopeptidases and intracellular peptidases have a wide range of specificity, allowing them various proteolytic activities [29]. Owing to the use of bamboo shoots during the fermentation of beef, Cangkuk is a lactic acid fermentation product. The specificity of microbial enzymes from lactic acid bacteria in fermented beef enables production of two ACE inhibitory peptides which possess antihypertensive effects in SHRs.

The water-soluble fraction of Cangkuk demonstrated antihypertensive effects on SHRs after oral administration at a dose of 100 mg/kg BW. SBPs decreased by 14.9 mmHg after 8 h and showed an antihypertensive effect that was reduced after 24 h. The magnitude of the antihypertensive activity is directly proportional to the dose of the water-soluble fraction administered. The higher the dose, the greater the decrease in SBPs. The water-soluble fraction of Cangkuk has a long-term antihypertensive effect. However, meat protein hydrolysate of spent hens, hydrolyzed using pepsin and a combination of pepsin and pancreatin, showed an antihypertensive effect on SHRs after oral administration at 200 mg/kg BW. Spent hen hydrolysate-pepsin and spent hen hydrolysate-pepsin+pancreatin could decrease SBPs by 26.5 mmHg and 35.6 mmHg, respectively, with a maximum reduction in SBP after 2 h. The antihypertensive effect of the hydrolysates was reduced after 8 h and completely lost at 24 h [30].

ACE-inhibitory peptides that possess antihypertensive effects are a primary target in exploration for alternative natural antihypertensive agents. Both ACE-inhibitor peptides from Cangkuk (EAPLNPKANR and IVG) could decrease blood pressure in SHRs 8 h after administration of the peptides. The tripeptide IVG significantly lowers SHRs blood pressure and to a greater degree than the decapeptide EAPLNPKANR. Presumably, the smaller peptide (tripeptide) and the presence of isoleucine at the N-terminal had a propensity to enter and bind to active sites of ACE, and inhibit angiotensin II formation. Therefore, it could decrease blood pressure [5].

This study is comparable to one in which three novel peptides were identified from *Paralichthys olivaceus* surimi. Tetrapeptide IVDR exhibited high ACE inhibitory activity and could decrease blood pressure in SHRs 6 h after its administration [31]. This indicates that the presence of the amino acids, isoleucine and valine in this sequence, plays an important role in the mechanism of reducing blood pressure in SHRs. There is strong association between ACE inhibition and the antihypertensive effects of a bioactive peptide owing to its structure, size, chain length, type and order of amino acids in the sequence [32]. Bromelain hydrolysate of rabbit meat protein was used to generate a novel ACE-inhibitory peptide, WGAP, that exhibited antihypertensive activity when orally administered at 100 mg/kg BW in SHRs. WGAP could reduce the SBP in SHRs by up to 42.6 mmHg 4 h after oral administration [33].

A potent antihypertensive effect has also been reported for another meat product, Spanish dry-cured ham. The antihypertensive effects of its water-soluble fractions could reduce SBP by up to 38.3 mmHg when orally administered to SHRs [12]. Our research shows that the antihypertensive effects of the water-soluble fraction of Cangkuk were less than that of meat protein hydrolysate and cured meat (dry cured ham), but it could still give a long-term antihypertensive effect in SHRs. It is possible that the decapeptide EAPLNPKANR and tripeptide IVG can be categorized as food-derived antihypertensive peptides, produced by spontaneous fermentation of beef meat with addition of bamboo shoots. Traditional fermented beef meat (Cangkuk) from Indonesia was produced under a spontaneous fermentation and it has great potential as a functional food in terms of an antihypertensive food agent.

## Figures and Tables

**Table 1:** Amino acid sequence, IC_50_ value and position in parent proteins

Amino acid sequence	IC_50_ value (μM)	Position in parent proteins and source
EAPLNPKANR	44.6	22nd to 31st residues of β-actin of goat meat protein [25] 107th to 116th residues of actin gamma 1 of Bovine (Hybrid cattle) (Uniprot ID: Q5E9B5)
IVG	97.3	Dried bonito [26] 34th to 36th residues of actin gamma 1 of Bovine (Hybrid cattle) (Uniprot ID: Q5E9B5) Chicken Muscle Protein (Actin) [26] Beef myofibrillar protein [8]

IC_50_, half maximal inhibitory concentration.
